# Eczema of the Hand

**Published:** 1879-09-20

**Authors:** John Kent Spender


### *THE TREATMENT OF ECZEMA OF THE HAND, OFTEN MISCALLED PSORIASIS PALMARIS.*

BY JOHN KENT SPENDER, M.D.

How I wish that our masters and teachers of dermatology would make it plain beyond all cavil that there is no such thing as *psoriasis palmaris*, except as a syphilide. Dr. Liveing approaches the nearest to this decision of utterance when he writes (Handbook on the Diagnosis of Skin-Diseases) that "in syphilitic psoriasis, the palms and soles are often affected, but never in simple psoriasis." But, if the student or junior practitioner turn to the lamented Dr. Tilbury Fox's splendid Atlas of Skin Diseases, he will find a plate adopted from Willan & Bateman, and described as *psoriasis palmaris*, a "very obstinate form of psoriasis." It is true that Dr. Fox admits "a scaly thickened condition, with more or less fissuring of the palms of the hands and soles of the feet," as liable to follow eczema and some other affections; but he says that it may be also a part of general psoriasis,, which has traveled on to the palm of the hand from neighboring portions of skin.

Acknowledging that the circumferential tracts of the palm may be affected with true psoriasis, which has extended from the back of the hand, I confidently affirm (with the greatest respect for Dr. Tilbury Fox's learning and experience) that the disease represented in Willan & Bateman's plate is not psoriasis at all. The anatomical and physiological affinities of the skin of the palm forbid such an idea. The thing may look like psoriasis, but that is quite a different thing, as Dr. Fox would have been the first to admit. Fissures and moist scales on the flexor aspect of a limb proclaim unmistakably that a disease belongs to the eczematous group.

Dr. Fairly Clarke, in the Practitioner, has observed the confusion which arises from applying the term psoriasis to many different morbid conditions of the tongue. Dr. McCall Anderson describes the eczema of the hand of which I am now speaking, as *eczema rimosum*.

The practical point is this. Wrong names affixed to diseases of the skin suggest and invite wrong treatment. Eczema of the palm of the hand is so disguised and altered by the thickness of the dermal structures, that it is hard to believe the heaped-up, fissured, and often bleeding epidermis to be an eczematous affection. But it is certain that any application of an irritating nature will exasperate the disease, and anything of a specifically soothing nature will gradually cure it.

A gentleman a little past middle age, of a healthy constitution, and engaged in the civil service, came to Bath from London last autumn with an eczema of the hands and feet more intense than, I think, I had ever seen before. He had been under the care of a distinguished London surgeon, who had called it eczema, but had certainly treated it as a psoriasis; for *liquor carbonis detergens* had been prescribed in a lotion, and arsenic had been given internally. The result was most disastrous. No treatment with thermal waters could permanently benefit such useless hands and painful feet. The hands were alternately bathed with glycerine and covered completely with compound lead ointment (the form I always use is given in Mr. W. Spencer Watson's book on Diseases of the Nose and its Accessory Cavities), and light thread gloves were constantly worn. Only the ointment was applied to the feet. At a later stage, the parts were washed with milk and sulphur-soap; and towards the end of the treatment arsenious acid was ordered in the form of pills. At the expiration of about three weeks, my patient left Bath much better; he got through the terrible winter without serious drawback, and in February he was virtually well. For some time, he used the ointment occasionally; the smoothness and flexibility of the palms of the hands are perfectly restored, and he can walk any reasonable distance with ease and comfort.—*British Medical Journal.*

